# Adsorptive-Oxidative Removal of Sulfides from Water by MnO_2_-Loaded Carboxylic Cation Exchangers

**DOI:** 10.3390/ma13225124

**Published:** 2020-11-13

**Authors:** Łukasz J. Wilk, Agnieszka Ciechanowska, Elżbieta Kociołek-Balawejder

**Affiliations:** Department of Industrial Chemistry, Wroclaw University of Economics and Business, 118/120 Komandorska St., 53-345 Wroclaw, Poland; agnieszka.ciechanowska@ue.wroc.pl (A.C.); elzbieta.kociolek-balawejder@ue.wroc.pl (E.K.-B.)

**Keywords:** hybrid ion exchanger, manganese(IV) oxide, residual sulfides, sulfide removal, reductive dissolution

## Abstract

Hybrid ion exchangers (HIX) containing manganese(IV) oxide (MnO_2_) based on macroporous and gel-type carboxylic cation exchangers as supporting materials were obtained. The hybrid materials were characterized using scanning electron microscopy with energy dispersive spectrometry (SEM/EDS), Fourier transform infrared spectroscopy (FTIR), X-ray powder diffraction (XRD) and nitrogen adsorption isotherms at 77 K and mercury porosimetry. HIX with introduced MnO_2_ (20.0–32.8 wt% Mn) were tested for removal of dissolved sulfides from anoxic aqueous solutions with 100–500 mg S^2−^/dm^3^ concentrations. The process proceeded effortlessly at pH 10–13 despite unfavorable electrostatic interactions of the reactants. The highest exhibited sorption capacity was 144.3 ± 7.1 mg S^2−^/g. Approximately 65% of dissolved sulfides were oxidized to S_2_O_3_^2−^ ions and repelled from HIX structure. On average, 13% of sulfide removal products were adsorbed by the MnO_2_ surface. The impact of MnO_2_ load and the ionic form of HIX functional groups on removal of sulfides and resulting products was examined. The mechanism of the process is suggested.

## 1. Introduction

Sulfides (H_2_S_(aq)_, HS^−^, S^2−^) are known as unwanted and troublesome constituents of natural waters and diverse aquatic environments. This results from the ease of their formation in the environment and specific physicochemical properties. Sulfides can originate from widespread, commonly occurring, natural anaerobic processes—for instance, the reduction of SO_4_^2−^ ions assisted by sulfate-reducing bacteria, and degradation of organic matter containing sulfur atoms in its structure. These processes take place in an uncontrolled manner in marine, lake and river sediments as well as during transportation and storage of domestic, municipal or agricultural wastes and wastewaters. Sulfides can also be formed as byproducts of different large volume industrial processes, inter alia, refining of petroleum products, processing of coke and steel, pulp and paper production or mining [1,2,3,4,5,6,7,8].

There are many relevant adverse effects of sulfide presence in aquatic environments. In particular, even at negligible levels sulfides give water an obnoxious smell and taste, making it useless as drinking water or an ingredient/reactant in the food and beverage industry. Sulfides react spontaneously with metals and metal oxides, causing corrosion of steel pipelines, storage tanks and heat exchangers, which is especially harmful when nickel is used as an alloying component. If metal or metal oxides are the active components of catalysts, the presence of sulfides in the reaction medium usually causes their poisoning and deactivation. Sulfides can be easily, and in a wide pH range, emitted in the toxic form of H_2_S_(g)_. Therefore transportation, processing and treatment of sulfide-containing solutions represent a threat to human health and life or, at the very least, a serious odor nuisance. Moreover, the emitted H_2_S can react with alkaline components of concrete, being the reason for gradual decay of its structure. Hydrogen sulfide initiated concrete corrosion is known to cause severe damage to municipal sewer systems, which is associated with incurring significant costs for their maintenance [9,10,11,12,13].

The adverse effects of sulfides and their negative impact on the surrounding environment can be decreased by several methods. The most commonly applied are aeration of reaction media, sulfide oxidation by addition of low-molecular weight oxidants (such as O_2_, O_3_, H_2_O_2_, Cl_2_, ClO_2_) or their precipitation to form FeS using iron salts. These strategies are effective enough to oxidize or precipitate the majority of sulfides present in treated solution. However, they do not (and are not designed to) neutralize sulfides completely. The residual sulfides left in the solution can be removed using various adsorption techniques, which is considered to be a forward-looking solution [11,14,15,16].

Among the adsorbents suitable and technically important for sulfide removal from different environments are iron(III) oxides, which are considered as cheap, non-toxic and thermodynamically stable reactants [17,18,19,20,21,22,23]. In one of our research papers [24], we reported that the drawbacks resulting from using powdered iron(III) oxide, in particular decrease in surface area due to aggregation, poor mechanical strength and separation of iron oxide from the solution, could be avoided by depositing the oxide in the inner structure of a cation exchanger. The hybrid ion exchangers (HIX) obtained using gel-type and macroporous carboxylic cation exchangers as the host materials had a high overall iron oxide load (30–35 wt% Fe), which was correlated with a considerable sulfide removal capacity (max. 60 mg S^2−^/g). The described process was complicated and proceeded through iron(III) oxide reductive dissolution, oxidation of sulfides and formation of their different precipitation products. Moreover, the form of carboxylic functional groups (Na^+^ or H^+^) of the sorbent had a crucial impact on the sulfide removal process.

In the present study, we deposited manganese(IV) oxide (MnO_2_) in the structure of tested commercial gel-type and macroporous carboxylic cation exchangers. The MnO_2_ was selected since it is the strongest and most abundant known oxidizer in natural systems after oxygen [25,26], and has been widely used, as fine or ultrafine particles, for oxidation/removal of toxic substances from aquatic environments [27,28,29,30,31,32,33,34]. Moreover, MnO_2_ has been successfully introduced into the structure of different carriers—for instance cellulose, biochar, chitin, chitosan, alginate beads, activated carbon, carbon fibers and nanotubes, graphene, polyester fiber, ion exchange resins, as well as poly- (vinyl alcohol) and (vinyl pyrrolidone). The obtained materials have been exploited as versatile sorbents for, inter alia, Cd^2+^, Cu^2+^, Li^+^, Ni^2+^, Pb^2+^, Sr^2+^, Zn^2+^, As(III), As(V) and anionic dyes from water [35,36,37,38,39,40,41,42,43,44,45,46,47,48,49,50,51,52,53]. We chose a simple HIX synthesis method which is based on ion exchange reaction of carboxylic functional groups with Mn^2+^ followed by an oxidation/disproportionation reaction in the presence of MnO_4_^−^.

The aim of this study was to obtain and characterize hybrid materials with different MnO_2_ load and form of functional groups, evaluate their effectiveness for sulfide removal, compare the products of the reactions and suggest a mechanism of examined processes. To the best of the authors’ knowledge, HIX containing MnO_2_ obtained using cation exchangers as hosts have never previously been tested for removal of sulfides from water. Such hybrid materials should combine the oxidation ability of MnO_2_ with a large surface area, well-developed porous structure and great hydrodynamic properties of cation exchangers. Moreover, cation exchangers are commercially available worldwide which allows the study to be easily reproduced.

## 2. Materials and Methods 

The polymeric support for MnO_2_ was cation exchangers—Amberlite IRC50 and Amberlite IRC86, commercial products sourced by The Dow Chemical Co. (Midland, MI, USA) The physicochemical properties of polymeric base materials are described in Table 1. 

Samples of Amberlite IRC 50 and Amberlite IRC 86 (10.0 g, air-dry condition) were mixed on a laboratory flat shaker (IKA, KS 260 Control, Staufen, Germany) in transparent non-stoppered conical flasks with 1 M NaOH/1 M NaCl in order to change the H^+^ ionic form of the carboxylic functional groups of the resins to Na^+^. Reactions were carried out providing 100% molar excess of Na^+^ in relation to the amount required by the stoichiometry. After 24 h, samples were washed with distilled water to obtain the neutral pH of the solution and dried at 40 °C for 24 h in a chamber laboratory dryer. In the next step, samples were mixed with 0.2 M MnCl_2_ solution ensuring a 100% molar excess of Mn^2+^ cations in relation to the stoichiometry. The process of ion exchange was carried out for 24 h, then samples were rinsed with distilled water until the Cl^−^ ions were undetectable in the filtrate and dried as before. In order to oxidize the Mn^2+^ ions, a solution of 0.2 M KMnO_4_ was used (in the molar ratio ensuring 100% molar excess of the MnO_4_^−^ ions in relation to the stoichiometry). After 24 h, samples were rinsed with distilled water and ethanol until the MnO_4_^−^ ions were undetectable in the filtrate, and dried at 40 °C for 24 h. A part of the obtained HIX underwent the whole cycle of synthesis twice again. All the chemical reagents used were of analytical grade (Chempur, Piekary Śląskie, Poland).

The methods and equipment used for: (1) characterization of obtained hybrid materials using scanning electron microscopy with energy dispersive spectrometry (SEM/EDS, Hitachi, Tokyo, Japan), Fourier transform infrared spectroscopy (FTIR, Bruker, Billerica, MA, USA), X-ray powder diffraction (XRD, Rigaku, Tokyo, Japan), nitrogen adsorption isotherms at 77 K and mercury porosimetry techniques (Micromeritics, Norcross, GA, USA); (2) the preparation of sulfide solutions and procedures of adsorption experiments; (3) the analysis of total sulfur and manganese content in HIX structure and aqueous phase; as well as (4) the determination of sulfide, sulfate and thiosulfate ions in examined solutions are described in detail in our previous research papers [24,54]. In order to enable others to reproduce our results, the description of the methods and procedures is also included as a Supplementary Material (detailed description of the Methods Section). 

## 3. Results and Discussion

Considering the need for innovative reactants and methods for removal of sulfides from aqueous solutions, we decided to undertake such research for carboxylic cation exchangers containing MnO_2_ in their polymeric matrix. The host materials were received with functional groups in hydrogen form. Since H^+^ ions have greater affinity for weakly acidic carboxylic groups than Mn^2+^ ions, in order to incorporate Mn^2+^ cations in the polymer structure, a first, preliminary stage of the synthesis process was required. In this stage, cation exchange resins were contacted with a solution of NaOH/NaCl, which caused an ion exchange reaction between carboxylic groups in H^+^ form and Na^+^ ions (1):

[P]-COO^−^ H^+^ + Na^+^ → [P]-COO^−^ Na^+^ + H^+^ (1)

The obtained cation exchangers in Na^+^ form, following a series of affinity, underwent ion exchange reaction with Mn^2+^ ions (2) in the second stage of the process:

[P]-(COO^−^ Na^+^)_2_ + Mn^2+^ → [P]-(COO^−^ )_2_Mn^2+^ + 2 Na^+^ (2)

Then the Mn^2+^ ions were oxidized by KMnO_4_ during the third stage of the process, which could be described by a simplified Reaction (3):

[P]-(COO^−^ )_2_ Mn^2+^ + x(K^+^ + MnO_4_^−^) → [P]-(COO^−^ K^+^)_x_ #MnO_2_ [P]-poly(acrylic-divinylbenzene) matrix, #—within the matrix(3)

The method used for obtaining hybrid cation exchangers was described previously in the work of Su et al. [51] and modified by Wang et al. [55]. The change made by Wang et al. was to use KMnO_4_ instead of NaOCl as the oxidant during the synthesis process. In order to ensure high uniformity of the obtained product, the introduction of MnO_2_ into the copolymer matrix was performed using the batch method.

Hereinafter, the cation exchange resins with carboxylic weakly acidic functional groups ([P]-COO^−^) having macroporous (Amberlite IRC50) or gel-type (Amberlite IRC86) matrix structure are referred to as CWM and CWG. The obtained hybrid materials containing MnO_2_ within the polymer matrix ([P]-COO^−^#MnO_2_) are referred to as CWM#MnO_2_ and CWG#MnO_2_, respectively. Where necessary, the ionic form of functional groups was added to the term after a slash (/), for instance CWM/Na^+^ or CWM#MnO_2_/Na^+^. To indicate the number of synthesis cycles performed, an Arabic numeral was added, for example CWM1#MnO_2_ or CWM3#MnO_2_.

The content of manganese dispersed in the HIX structure (by weight, ICP-AES) was 20.0%, 32.8%, 18.9% and 22.1% for CWM1#MnO_2_, CWM3#MnO_2_, CWG1#MnO_2_ and CWG3#MnO_2_, respectively.

The process of introduction of the inorganic deposit into the structure of cation exchangers caused changes in the appearance of their beads. The beads of CWM#MnO_2_ and CWG#MnO_2_ were black and had a navy-blue luster; the appearance of their polymeric precursors is described in Table 1. Apart from the color, there were no visible changes of the beads, including any signs of cracking or peeling.

All synthesized hybrid materials were preliminarily tested to confirm their capacity for removal of sulfide species from aqueous solutions. For this purpose, 0.50 g of HIX was mixed with 100 cm^3^ of 100 mg S^2−^/dm^3^ sulfide solution for 24 h. In the case of CWM#MnO_2_, sulfides were completely removed from the solution. CWG1#MnO_2_ and CWG3#MnO_2_ removed 61% and 57% of sulfides, respectively. The removal efficiency of CWG#MnO_2_ was considerably lower compared to similar hybrid materials with iron(III) oxides described in our previous work [24]. Therefore, for further examinations, only hybrid materials obtained from a cation exchanger with a macroreticular matrix were chosen.

### 3.1. Characterization

**Figure 1:**
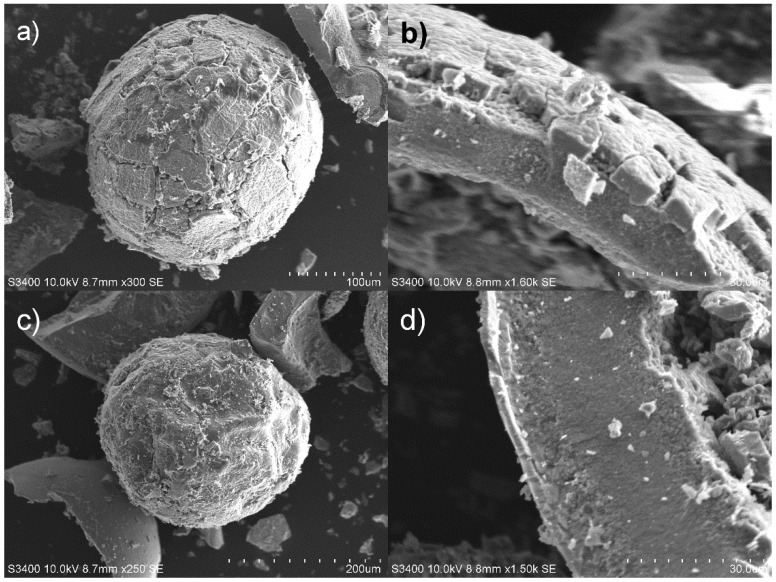
SEM images of hybrid materials: (**a**) CWM1#MnO_2_ bead’s outer surface, (**b**) structure of CWM1#MnO_2_ surface layer, (**c**) CWM3#MnO_2_ bead’s outer surface, (**d**) structure of CWM3#MnO_2_ surface layer.

The distribution of MnO_2_ within HIX beads was studied using the SEM/EDS technique. Prior to microscopic examinations, the beads were lightly crushed in a ceramic mortar, which revealed their inner structure and simultaneously damaged them, as can be seen in Figure 1.

**Figure 2:**
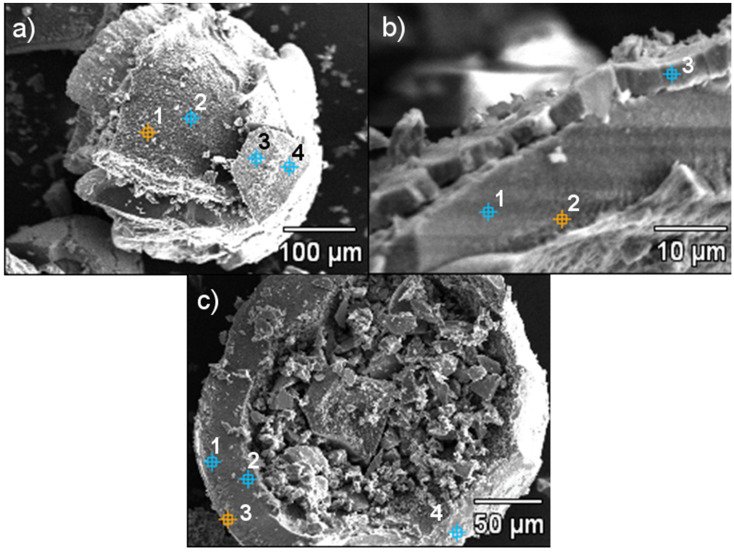
Areas of hybrid ion exchangers (HIX) examined using EDS point analysis: (**a**,**b**) CWM1#MnO_2_, (**c**) CWM3#MnO_2_.

SEM images revealed that HIX beads had an irregular rough surface with visible cracks. Moreover, the surface had a multilayer structure (Figure 1b,d) formed by gradually depositing MnO_2_ oxide. The EDS point analysis carried out for selected areas of HIX beads (Figure 2, Table 2) showed that the outer layer of CWM1#MnO_2_ (Figure 2, points 3 and 4) had a significant Mn content, whereas Mn was not detected in the deeper structure of the beads (Figure 2, points 1 and 2). Therefore, the formation of the MnO_2_ layer took place at the outer surface of CWM beads (similarly to in the work of Lenoble et al. [56]), making the polymeric support a core, and the inorganic deposit a shell of synthesized hybrid material.

Since sodium was detected at all points of EDS analysis of CWM1#MnO_2_, the formation of MnO_2_ blocked sodium ions in carboxylic functional groups, which suggests that the ion exchange reaction in the Na^+^/Mn^2+^ system was incomplete. The formation of the inorganic shell probably took place, light and air induced, simultaneously with the mentioned ion exchange reaction and continued during the addition of KMnO_4_ solution, which could explain the surface consisting of two MnO_2_ layers seen in Figure 1b and Figure 2b. The presence of potassium was a result of using KMnO_4_ in the HIX synthesis process. Sodium was not detected in the EDS analysis of CWM3#MnO_2_, which suggests that during the three cycles of synthesis, the Na^+^ ions were completely replaced by Mn^2+^ and K^+^ ions from KMnO_4_. Oxygen detected in all performed EDS analyses indicates that Mn in hybrid material beads was introduced in the form of an oxide and/or hydrated metal oxide.

**Figure 3:**
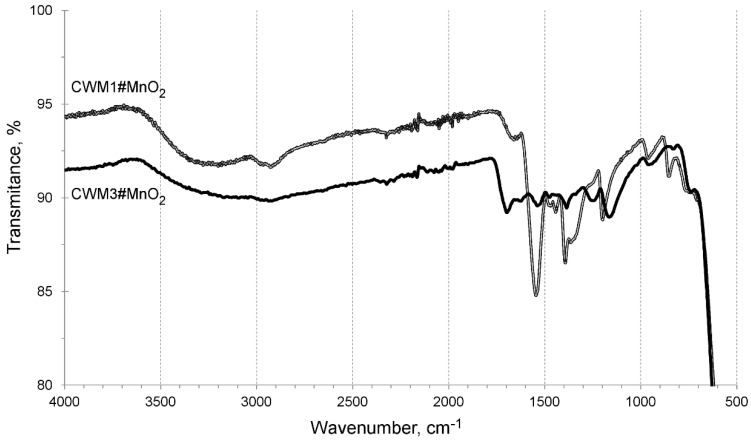
FTIR spectra of CWM#MnO_2_ in medium infrared range.

In order to examine the organic–inorganic interactions of the constituents of obtained HIX, infrared spectroscopy was used. FTIR spectra of hybrid materials in the medium infrared (MIR) range, after one and three cycles of synthesis, are shown in Figure 3. They consist of two main sets of bands: (a) 3700–2500 cm^−1^ characteristic for different O-H interactions, and (b) 1600–750 cm^−1^, which can be attributed mostly to the hydrocarbon polymeric matrix and its functional groups. 

The absorption bands in the wide range of 3700 to 2500 cm^−1^ can be described as a superposition of different stretching and bending vibrations of O-H bonds. The vibrations in the range of 3700–3600 cm^−1^ might indicate the presence of free hydroxyl groups, in the range 3500–3200 cm^−1^ of hydrogen bonded hydroxyl groups and in the range 3300–2500 cm^−1^ of O-H interactions in carboxylic functional groups. The stretching and bending O-H vibrations are normal for hybrid materials containing metal and hydrous metal oxides or hydroxides. There is also a strong possibility of presence of adsorbed water in examined HIX beads. Vibrations in the range 1600–1500 cm^−1^ with a visible absorbance peak at ~1550 cm^−1^ can be assigned to C=O bonds in –COO^−^ functional groups. The adsorption band in the range 1500–1400 cm^−1^ could be attributed to vibrations of aromatic rings in polyacrylic-divinylbenzene copolymer and scissoring vibrations of C-H bonds. The band in the range of 1400–750 cm^−1^ could be assigned to overlapping: bending and wagging vibrations of C-H bonds, stretching C-O and bending O-H vibrations, as well as stretching C-C and C-H bond vibrations [57,58,59,60,61,62]. There were no characteristic bands in the far infrared (FIR) range.

The FTIR spectra of CWM1#MnO_2_ and CWM3#MnO_2_ are similar in the range of 3900 to 1800 cm^−1^ since this band range is assigned to different vibrations of O-H bonds. We suggest that a significant part of –OH groups could be coupled with manganese in the inorganic component of examined sorbents. The flattening of the CWM3#MnO_2_ spectrum in the range of 1800–600 cm^−1^ was probably a result of covering of the organic matrix by layers of MnO_2_ deposit coupled with degradation of its hydrocarbon structure by KMnO_4_. Moreover, the CWM3#MnO_2_ hybrid material had no visible absorbance peak in the range of 1600–1500 cm^−1^, which suggests that it lost most of its carboxylic functional groups and ion exchange ability.

**Figure 4:**
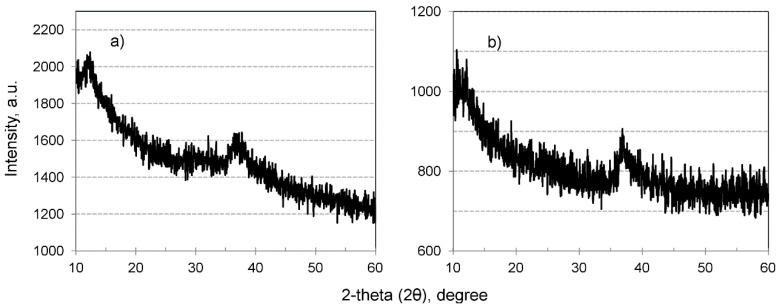
XRD patterns of hybrid material: (**a**) CWM1#MnO_2_, (**b**) CWM3#MnO_2_.

In order to examine the structure of MnO_2_ in HIX beads, X-ray powder diffraction analyses were performed. The obtained XRD patterns (Figure 4) suggest that the inorganic deposit was amorphous for both the CWM1#MnO_2_ and CWM3#MnO_2_. It is noteworthy that the peak for a 2-theta angle of 37 is used for identification of hausmannite (γ-Mn_3_O_4_, ICSD no. 68174) [63]. However, there were no other visible reflections of MnO_2_, which could have been covered by a high background of organic component of examined materials. 

The porous structure of the sorbents was examined using adsorption/desorption of nitrogen in 77 K, and mercury porosimetry. A nitrogen adsorption technique was applied to determine pore texture in the range of 0.7–50 nm. Mercury porosimetry was used for examination of partially complementary and wider pore structure, specifically in the range of dimensions from 3 to 600 nm. The obtained data are reported in Table 3 and Table 4.

The majority of the CWM polymeric precursor inner structure was composed of pores exceeding 50 nm in diameter, which is typical for macroreticular ion exchangers. During the first cycle of synthesis, a significant decrease in pore texture in the range of 3–600 nm took place. The changes of pore texture in the range of 0.7–50 nm were negligible, but a slight decrease in Brunauer-Emmet-Teller (BET) surface area was visible. This phenomenon could be explained by deposition of MnO_2_ in the pores and near the surface of CWM raw material beads. The formed layer of inorganic deposit (Figure 1b) was coherent enough to hinder the intrusion of mercury into the hybrid material inner structure.

**Figure 5:**
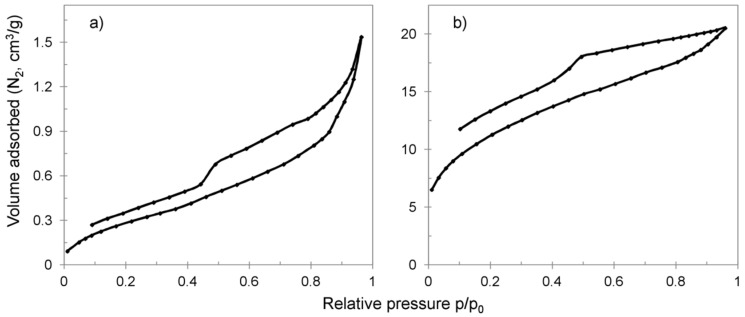
Nitrogen adsorption/desorption isotherms for hybrid materials: (**a**) CMW1#MnO_2_, (**b**) CWM3#MnO_2_.

Hybrid material after three cycles of synthesis had a well-developed mesoporous structure compared to CWM and CWM1#MnO_2_—BET surface area of 40 compared to 1.67 and 1.15 m^2^/g, respectively. During the second and third cycle of synthesis, the total surface area in the partially complementary pore texture of 3–600 nm also increased from 3.45 to 18.29 m^2^/g. We suggest that the significant increase in the overall surface area was a result of formation of a multilayer porous MnO_2_ shell on the polymeric core, which was revealed during SEM/EDS examinations. The possible presence of pores wider in diameter than 50 nm in that shell could be attributed to cracks, peeling and interlaminar gaps in deposited MnO_2_ surface structures. The existence of interlaminar gaps may be confirmed by the shape of the hysteresis loop of CWM3#MnO_2_ (Figure 5b), which was open and of type H4. The hysteresis loop of CWM1#MnO_2_ was open and of type H3, which could indicate that the pore structure was filled with MnO_2_ and that the pore inner diameters could have been bigger than their entrances [64,65,66,67].

The changes of apparent density that occurred during the synthesis process were also interesting and could confirm some of our suggestions. An increase in density was noticed after the first cycle of MnO_2_ introduction into the CWM matrix, whereas there was no considerable change of that parameter between CWM1#MnO_2_ and CWM3MnO_2_. This could indicate that at first MnO_2_ was deposited in the inner structure and near the surface of CWM, which partially blocked its pore structure and simultaneously considerably altered the density of beads. Therefore, subsequent layers of MnO_2_ could be deposited only on the limited sorbent surface, which did not induce such noticeable changes of density.

### 3.2. Chemisorption Studies

**Figure 6:**
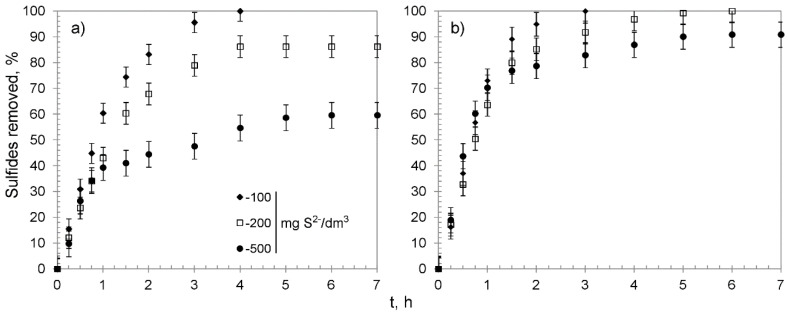
Sulfide removal effectiveness of hybrid materials versus time: (**a**) CWM1#MnO_2_, (**b**) CWM3#MnO_2_; on average, n = 3.

HIX, with functional groups in the ionic state in which they were synthesized, were tested for sulfide removal effectiveness during kinetic chemisorption studies. Kinetic chemisorption studies were performed using 0.30 g of the HIX for solutions containing 100, 200, and 500 mg S^2−^/dm^3^. The obtained results are shown in Figure 6.

The CWM1#MnO_2_ removed all sulfide species from the solution with concentration of 100 mg S^2−^/dm^3^. In the case of more concentrated solutions, the removal efficiency reached 86.2 ± 4.2% and 59.6 ± 5.0%. That corresponded to the average removal capacity after the reaction reached an equilibrium (q_e_) of 54.8 ± 0.23 and 94.7 ± 4.8 mg S^2−^/g for solutions containing 200 and 500 mg S^2−^/dm^3^, respectively (for greater clarity and comparability, all relevant data for every examined sorbent are summarized in Table 5). The CWM3#MnO_2_ completely purified solutions containing 100 and 200 mg S^2−^/dm^3^, whereas from the solution with the highest tested S^2−^ concentration, over 90% of sulfides were removed. The overall removal capacity of CWM3#MnO_2_ amounted to 144.3 ± 7.1 mg S^2−^/g and was on average about 50 mg S^2−^ higher than the q_e_ of CWM1#MnO_2_. The process could be divided into three stages (Figure 6): (1) a relatively fast stage of sulfide removal, probably by MnO_2_ surface active sites, during 1–2 h; (2) infiltration of sulfide species into deeper layers of the sorbent between 2 and 4 h; (3) reaching an equilibrium after 4–5 h, which suggests saturation of MnO_2_ active sites. The slope of the kinetic curves indicates that CWM3#MnO_2_ had a higher removal rate during the first stage than its counterpart after one cycle of MnO_2_ deposition.

**Figure 7:**
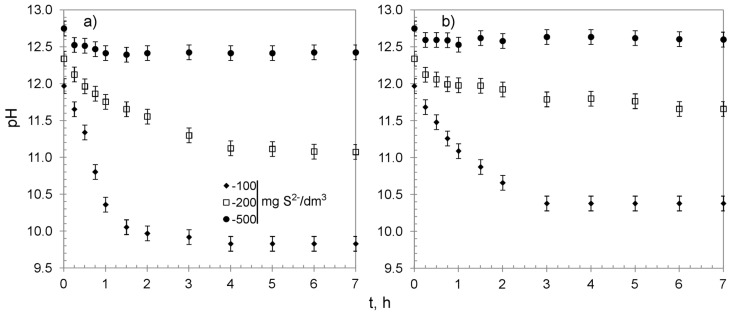
Changes of pH in treated solutions versus time: (**a**) CWM1#MnO_2_, (**b**) CWM3#MnO_2_; on average, n = 3.

The process of sulfide removal from water proceeded with a decrease in the solutions’ pH (Figure 7). The changes occurred during the first 3–4 h, accompanied by the removal of the majority of sulfides, after which pH nearly reached a plateau. That could be explained by the fact that sulfides were completely removed after 4 h from the solution containing 100 mg S^2−^/dm^3^. Simultaneously, in that time, both sorbents reached over 95% of their overall average removal capacity for solutions containing 200 and 500 mg S^2−^/dm^3^, and the process began to reach an equilibrium. It is worth noting that the changes of pH were more visible for CWM1#MnO_2_, which was probably caused by the presence of carboxylic functional groups (the FTIR analyses of CWM3#MnO_2_ revealed that it lost most of its functional groups during synthesis, Figure 3).

In our last study describing hybrid materials obtained from cation exchangers containing metal oxides, the H^+^ ionic form of functional groups had crucial importance for the sulfide removal process [24]. Therefore, in order to change the ionic form of carboxylic functional groups to H^+^, a part of CWM1#MnO_2_ and CWM3#MnO_2_ was mixed several times with 0.05 M CH_3_COOH. The ion exchange was considered complete when no changes of CH_3_COOH concentration and pH of the solution occurred. Afterwards, the sorbents were rinsed with distilled water (to obtain a neutral pH) and ethanol, and dried at 40 °C for 24 h. Since a weak, diluted organic acid was used to alter the ionic form of functional groups, the changes in Mn content in HIX beads were negligible.

**Figure 8:**
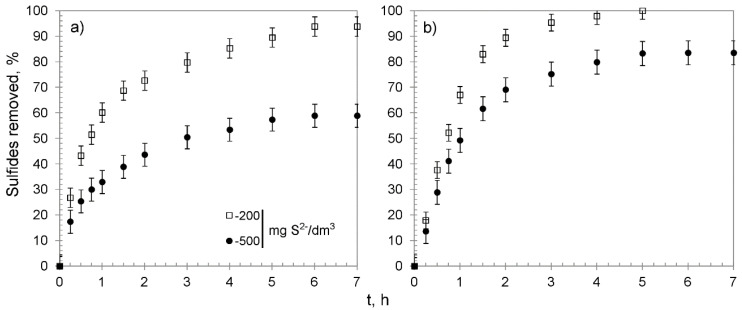
Sulfide removal effectiveness of hybrid materials versus time: (**a**) CWM1#MnO_2_/H^+^, (**b**) CWM3#MnO_2_/H^+^; on average, n = 3.

**Figure 9:**
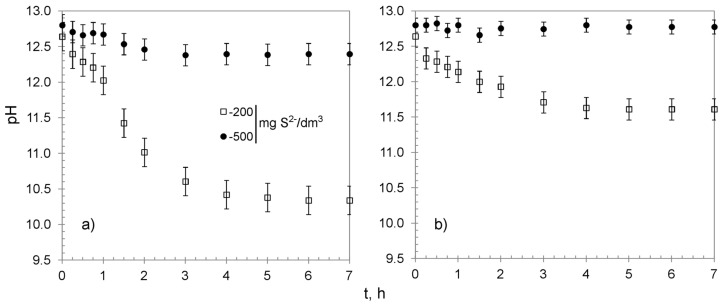
Changes of pH in treated solutions versus time: (**a**) CWM1#MnO_2_/H^+^, (**b**) CWM3#MnO_2_/H^+^, on average, n = 3.

The kinetic studies were performed using 0.30 g of CWM#MnO_2_/H^+^ and solutions containing 200 and 500 mg S^2−^/dm^3^. Their results are shown in Figure 8 and summarized in Table 5. The removal efficiency reached 93.8 ± 3.8 and 58.9 ± 4.5% of the sulfides for CWM1#MnO_2_/H^+^, which amounted to a value of q_e_ of 65.2 ± 2.5 and 99.0 ± 4.5 mg S^2−^/g for solutions with concentration of 200 and 500 mg S^2−^/dm^3^, respectively. The sulfides were completely removed by CWM3#MnO_2_/H^+^ from the solution containing 200 mg S^2−^/dm^3^, whereas the sulfide removal efficiency for the most concentrated solution reached 83.5 ± 4.7%, which corresponded to overall removal capacity of 140.4 ± 6.6 mg S^2−^/g. The analysis of kinetic curves suggests that the process could be divided into three stages, and that CWM3#MnO_2_/H^+^ had a higher removal rate than its counterpart after one cycle of synthesis—similarly to in the case of hybrid materials without the altered ionic form of functional groups. The removal of sulfides was accompanied by a decrease in solutions’ pH. The change was particularly evident for the solution having 200 mg S^2−^/dm^3^ concentration and CWM1#MnO_2_/H^+^ sorbent (Figure 9). This phenomenon was expected, since carboxylic functional groups in hydrogen form had the ability to reduce the pH as a result of the ion–exchange reaction (Na^+^ was a constituent of Na_2_S used to prepare the solutions):

[P]-(COO^−^ H^+^)#MnO_2_ + Na^+^ → [P]-(COO^−^ Na^+^)#MnO_2_ + H^+^ (4)

As mentioned previously, CWM3#MnO_2_ lost most of its ion exchange ability, and therefore, the Reaction (4) had a minor or no impact on the pH. 

In order to understand and describe the mechanism of sulfide species removal using CWM#MnO_2_, the products of chemical reactions occurring in examined systems were quantified. As can be seen in Table 5, the main product of sulfide removal was thiosulfate ions. In the case of hybrid material after one cycle of synthesis (with or without modified ionic functional groups), the S_2_O_3_^2−^ ions accounted for about 70–90% of sulfides removed overall, whereas only up to about 6% (mostly less than 4%) of the sulfides were bound to hybrid material beads. The S_2_O_3_^2−^ ions were also the main product of the process when CWM3#MnO_2_ was used, and accounted for about 60–75% of sulfides removed overall. However, in the case of CWM3#MnO_2_, simultaneously nearly 20% to 30% of the sulfides were bound to the structure of the beads. The process proceeded with the release of a part of the inorganic deposit from HIX beads into the aqueous phase of the examined systems (hereinafter referred to as the sediment). The manganese dissolved in the solution (or in particles smaller than 0.2 µm) contributed to less than 2% of Mn released overall. The amount of released manganese was greater the higher the concentration of treated S^2−^ solution was. However, the hybrid materials with carboxylic functional groups in H^+^ form were more durable—the amount of Mn in the solutions never exceeded 5% of Mn initially present in the sorbent beads. For instance, for solution containing 500 mg S^2−^/dm^3^, the amount of Mn released was 25.64% for CWM3#MnO_2_ compared to only 3.52% for CWM3#MnO_2_/H^+^. Notably, the amount of unbalanced sulfides removed was also positively correlated with the concentration of S^2−^ solution and the amount of Mn released, which might suggest that at least a part of those unbalanced sulfides was removed from the examined system as a constituent of the sediment

Hydrogen sulfide is a weak acid (pK_a1_ = 7.0, pK_a2_ = 13.0), and since the pH of all examined systems was in the range of about 10 to 13 (Table 5), the main form of sulfide species in the solution was HS^−^ anions [11,25]. The contact between hydrosulfite ions present in the aqueous phase and the structure of HIX beads was hindered because HS^−^ ions were repelled by electrostatic interactions of fixed carboxylic groups of the polymeric matrix (–COO^−^) according to the Donnan exclusion effect [68,69]. Moreover, the inorganic deposit in the mentioned pH range and in the case of hybrid materials without modified functional groups also had negative, Mn-O^−^, speciation (MnO_2_ exhibits pH_zpc_ of 4.6), which affected the transport of HS^−^ anions toward MnO_2_ surface active sites [25,26,70,71]. Nevertheless, the sulfides were removed by MnO_2_ by oxidation to S_2_O_3_^2−^, and in a form permanently bound to the HIX beads.

There are several possible mechanisms of interactions between MnO_2_ and sulfide species in an aquatic environment [25,26,72,73,74,75]. Based on obtained experimental data, we suggest that in the case of HIX without modified functional groups, despite electrostatic –COO^−^/MnO^−^:HS^−^ repulsion, a direct Mn-S surface complex was formed. The formation of such a complex is considered possible even in a strongly basic pH and consists of formation of a precursor (5) and a main surface complex (6) [25,26]:

≡Mn^(IV)−^O^−^ + HS^−^ → ≡Mn^(IV)^-O^−^—HS^−^ (5)

≡Mn^(IV)^-O^−^—HS^−^ → ≡Mn^(IV)^S^−^ + OH^−^ (6)

within the formed complex, donation of two electrons occurs with a S∙^−^ free radical being the possible intermediate product [75], elemental sulfur the final product, and a decrease in the Mn atom’s oxidation state in the MnO_2_ structure:

≡Mn^(IV)^S^−^ → ≡Mn^(III)^S^∙^ (7)

≡Mn^(III)^S^∙^ → ≡Mn^(II)^S^0^ (8)

In the case of hybrid materials with the modified H^+^ ionic form of carboxylic functional groups, the process could have been even more complicated. Firstly, the ion exchange Reaction (4) between mobile H^+^ ions and Na^+^ cations present in the Na_2_S solution could have facilitated the transport of HS^−^ anions toward the HIX surface. Secondly, because of the presence of H^+^ cations in the bead structure of CWM#MnO_2_/H^+^, there is a possibility that locally and temporarily the surface speciation of their MnO_2_ deposit was Mn-OH. Assuming that carboxylic groups in hydrogen form had the ability to locally decrease the pH and affect the MnO_2_ surface speciation, the formation of the Mn-S complex could be described by the reactions [25,26]:

≡Mn^(IV)−^OH + HS^−^ → ≡Mn^(IV)^-O—HS^−^ (9)

≡Mn^(IV)^-OH—HS^−^ → ≡Mn^(IV)^S^−^ + H_2_O (10)

However, the formation of Mn-S direct complexes according to Reactions (9) and (10), if occurred in this study, was negligible since the ionic form of HIX carboxylic functional groups was quickly changed to Na^+^ according to Reaction (4). 

The comparison of overall removal efficiency and removal of products of sulfides using HIX with and without the altered form of carboxylic groups shows that the form of the functional group is not significant for the examined process. Sorbents with functional groups in K^+^ and H^+^ form had good sorption capacities, which were about 55 and 95 mg S^2−^/g in the case of CWM1#MnO_2_/K^+^ and 65 and 99 mg S^2−^/g in the case of CWM1#MnO_2_/H^+^ for solutions containing 200 and 500 mg S^2−^/dm^3^, respectively. The obtained maximal sulfide removal capacities were considerably higher than the capacities of ferric and alum water treatment residuals (14 mg S^2−^/g) [76], granular ferric hydroxide (29 mg S^2−^/g) [23], ZnO (30 mg S^2−^/g) and CuO (68 mg S^2−^/g) hydrous metal oxides without a carrier [77] or hybrid material with iron(III) oxide as an inorganic deposit obtained using CWM (60 mg S^2−^/dm^3^) [24]. Since HIX after three cycles of synthesis had no or a minor number of functional groups able to undergo the ion exchange reaction, their influence is even less visible and hard to identify. Nevertheless, for the solution containing 500 mg, S^2−^/dm^3^ q_e_ of CWM3#MnO_2_ was calculated to be about 144 and 140 mg S^2−^/g for HIX without and with altered carboxylic groups, respectively. Obtained maximal sorption capacities for CWM3#MnO_2_ were comparable with HIX containing MnO_2_ obtained using the macroporous strongly basic anion exchanger with quaternary ammonium functional groups, which was able to remove 150 mg S^2−^/g [54], and lower than estimated sorption capacity of bio-derived porous graphitic carbon impregnated with MnO_2_ (approximately 525 mg S^2−^/g) [78]. 

The factor that considerably affected the effectiveness of sulfide removal and the type of resulting products was the amount of inorganic deposit incorporated into the structure of the polymeric carrier. The HIX with a higher MnO_2_ load had higher sorption capacities, which was especially visible during the treatment of solution containing 500 mg S^2−^/dm^3^. Moreover, significant growth of sulfide removal products bound to the HIX beads occurred when hybrid materials after three cycles of MnO_2_ incorporation were used. For instance, after the treatment of 500 mg S^2−^/dm^3^ solution with CWM1#MnO_2_/H^+^, only about 4% of sulfides removed were bound to its beads, whereas in the case of CWM3#MnO_2_, it was about 23%. A possible explanation for this phenomenon could be the fact that HIX after three cycles of synthesis had a high (32.8%) load of MnO_2_ deposited in a multilayer manner mostly on the surface of the beads. Such a manner of MnO_2_ placement could have provided a high availability of surface active sites where surface complexes were formed (according to Reactions (5)–(8)) with ≡Mn^(II)^S^0^ being the end product. The availability of active sites could have hindered the further oxidation and repulsion of S^0^ in ≡Mn^(II)^S^0^.

The S_2_O_3_^2−^ ions, which were the main product of sulfide removal regardless of the amount of MnO_2_ deposit (Table 5), could have been formed through oxidation of S^0^ bound in direct Mn-S complexes by neighboring active surface sites and repulsed to the solution by the –COO^−^/MnO^−^:S_2_O_3_^2−^ electrostatic interactions. The process of hydrosulfite ion formation can be described by a simplified Reaction (11):

≡(Mn^(IV)^O^−^)_x_ + ≡(Mn^(II)^S^0^)_x_ → ≡(Mn^(II)^O^−^)_x_ + S_2_O_3_^2−^ (11)

Although the presence of polysulfides was not analyzed in the present study, the decrease in solutions’ pH during the process (Figure 7 and Figure 9) of sulfide removal and favorable conditions (high pH and excessive amount of HS^−^ ions) might indicate their formation, for instance:

S^0^ + HS^−^ → S_2_^2−^ + H^+^ (12)

2S^0^ + HS^−^ → S_3_^2−^ + H^+^ (13)

3S^0^ + HS^−^ → S_4_^2−^ + H^+^ (14)

Moreover, polysulfides were detected on spent HIX beads containing dispersed MnO_2_ during a study performed in comparable conditions [54]. 

The Mn^2+^ ions that were formed in the examined systems according to Reactions (7) and (8) could have been not detached from the MnO_2_ in the HIX structure or partially detached from the HIX beads and resorbed afterwards by electrostatic interactions of the negatively speciated surface [72], as well as via ion exchange reaction with carboxylic functional groups. There is also a possibility that Mn^2+^ ions were repelled from the beads’ structure and formed Mn(OH)_2_ in the solution—the pH of Mn(OH)_2_ formation is 8.5–8.8, and Mn^2+^ ions do not react with excessive sulfides to form MnS↓ [25,75]. The Mn^2+^ ions could have also been present in the aqueous phase of examined systems as a constituent of the sediment (probably as parts of the inorganic deposit, specifically ≡Mn^(II)^S^0^). A possible ion exchange reaction between Mn^2+^ formed by a reduction in Mn atoms’ oxidation state in MnO_2_ structure and carboxylic functional groups, which was favorable according to cation series of affinity, is particularly interesting. Such a phenomenon could lead to self-regeneration understood as a process of recreation of MnO_2_ with the participation of HIX functional groups during the treatment of a contaminated solution.

## 4. Conclusions

HIX amorphous MnO_2_ oxide obtained using a commercial macroporous carboxylic cation exchanger was successfully used for removal of sulfide species from water. The content of inorganic deposit, expressed as the amount of Mn, incorporated into the polymeric precursor, was 20.0–32.8 wt%. The MnO_2_ was deposited mostly in the macroporous structure of the cation exchanger matrix, causing pore mouth narrowing, and then after on its beads’ surface in a multilayer manner. The deposition of MnO_2_ was accompanied by a considerable increase in bead density.

The examined sulfide removal process was complex and proceeded through a combination of adsorption and heterogenic oxidation. The main product was S_2_O_3_^2−^ ions, which accounted for 65% of sulfides removed on average. Simultaneously, on average, 13% of sulfide removal products were bound permanently to spent sorbent beads. The obtained maximal overall sorption capacity toward sulfides using synthesized sorbents was significant, reaching approximately 140 mg S^2−^/g. The key factor that had an impact on the process of sulfide removal effectiveness and type of resulting products was the amount of inorganic MnO_2_ deposit, whereas the influence of the ionic form of carboxylic functional groups was negligible in that matter. The oxidation and adsorption of sulfides were not hindered by unfavorable electrostatic interactions and proceeded effortlessly at pH 10.0–13.0. Since the process occurred without the need to lower the pH, the emission of sulfides in the form of toxic hydrogen sulfide from the solutions was not possible.

A part of the MnO_2_ deposit was released during the treatment of the solutions to the aqueous phase of examined systems. The sorbents with functional groups in H^+^ proved to be more resistant in terms of deposit durability. We suggest that this inconvenience is avoidable by choosing and incorporating the adequate quantity of MnO_2_ in polymer beads. It is also noteworthy that such a sorbent could have the ability to recreate the MnO_2_ by oxidation of Mn^2+^ formed during sulfide oxidation that was detached from the bead structure afterwards and resorbed via the ion–exchange reaction with carboxylic functional groups.

## Figures and Tables

**Table 1:** Characterization of polymeric base materials.

Property	Cation Exchanger	
	Amberlite IRC50	Amberlite IRC86
Matrix	poly(acrylic-divinylbenzene)	poly(acrylic-divinylbenzene)
Matrix structure	macroporous	gel
Functional groups	carboxylic	carboxylic
Ionic form	H^+^	H^+^
Exchange capacity ^1^, meq/g	10.8	10.7
Physical form	beads	beads
Diameter, mm	0.28–0.70	0.58–0.78
Appearance	creamy, opaque, matt	browhish-red, translucent, low sheen

^1^ In air-dry condition.

**Table 2:** Results of hybrid materials’ EDS point analysis.

Point	Content, wt.%			
	Mn	O	Na	K
CWM1#MnO_2_, Figure 2a				
1	-	36.9	15.8	0.7
2	-	37.1	18.0	0.8
3	36.8	39.0	7.5	1.3
4	31.9	37.3	8.2	1.0
CWM1#MnO_2_, Figure 2b				
1	41.0	32.0	6.5	1.0
2	48.2	21.7	6.8	1.3
3	50.0	34.8	5.5	1.5
CWM3#MnO_2_, Figure 2c				
1	40.3	34.8	-	5.6
2	40.3	38.6	-	4.7
3	48.9	38.1	-	5.0
4	69.9	6.4	-	3.0

**Table 3:** Porous characteristics of polymeric raw material and hybrid materials by N_2_ adsorption at 77 K.

Parameter	Sample		
	CWM	CWM1#MnO_2_	CWM3#MnO_2_
Brunauer-Emmet-Teller (BET) surface area, m^2^/g	1.67	1.15	40.03
BET total pore volume, cm^3^/g	0.0024	0.0024	0.0317
BET average pore diameter, nm	2.9	4.1	3.2
Barret-Joyner-Halenda (BJH) surface area, m^2^/g	1.61	1.79	29.51
BJH total pore volume, cm^3^/g	0.002	0.003	0.023
BJH average pore diameter, nm	1.68	3.74	3.81

**Table 4:** Porous characteristics of polymeric raw material and hybrid materials by mercury intrusion porosimetry.

Parameter	Sample		
	CWM	CWM1#MnO_2_	CWM3#MnO_2_
Total surface area, m^2^/g	25.70	3.45	18.29
Total porosity, %	7.73	8.17	14.29
Apparent density, g/cm^3^	1.157	1.739	1.781
Skeletal density, g/cm^3^	1.352	1.896	2.079
Total intrusion volume, cm^3^ Hg/g	0.124	0.047	0.080

**Table 5:** Mass balance of sulfide removal process with examined hybrid materials; on average, n = 3, RSD ≤ 5.0%

Hybrid Material	Sulfide Initial Concentration, mg S^2−^/dm^3^	Sulfides Removed: %				Manganese Released from the Beads to the Solution ^1^: %			pH of the Solution:		Overall Average Removal Capacity, mg S^2−^/g
		Overall	Bound to the Beads Structure	Present in the Solution in the S_2_O_3_^2−^ Form	Unbal-anced	Overall	Dissolved in the Solution	Bound in the Sediment	Initial	Final	
CWM1#MnO_2_/K^+^	100	100	4.0	89.7	6.3	0.32	0.17	0.15	12.0	9.8	34.1 ^2^
	200	86.2	3.4	76.7	6.1	2.24	0.52	1.72	12.3	11.1	54.8
	500	59.6	1.6	49.9	8.1	10.71	0.41	10.30	12.7	12.4	94.7
CWM3#MnO_2_/K^+^	100	100	20.8	75.1	4.1	1.98	0.32	1.66	12.0	10.4	34.1 ^2^
	200	100	28.4	61.5	9.9	10.82	1.85	8.97	12.3	11.6	63.3 ^2^
	500	90.9	15.8	60.9	14.2	25.84	0.21	25.64	12.7	12.6	144.3
CWM1#MnO_2_/H^+^	200	93.8	3.2	79.3	11.3	2.24	0.52	1.72	12.6	10.3	65.2
	500	58.9	3.6	40.3	15.0	5.37	0.63	4.74	12.8	12.4	99.0
CWM3#MnO_2_/H^+^	200	100	25.7	70.1	4.2	2.40	1.60	0.80	12.6	11.6	63.3 ^2^
	500	83.5	23.2	50.4	9.9	5.44	1.92	3.52	12.8	12.8	140.4

^1^ The methodology of manganese species quantification is described in detail in [54] and Supplementary Material (detailed description of the Method Section); ^2^ capacity calculated for the quantity of sulfides completely removed from the solution.

## Supplementary Materials

The following are available online at https://www.mdpi.com/1996-1944/13/22/5124/s1, detailed description of the Method Section.
