# Supplementary material for: Adsorptive-Oxidative Removal of Sulfides from Water by MnO2-Loaded Carboxylic Cation Exchangers
Source: Materials (Basel). 2020 Nov 13;13(22):5124. doi: 10.3390/ma13225124 (PMC7696700; doi:10.3390/ma13225124)
Supplement: Supplementary file 1 [file materials-13-05124-s001.pdf]

materials

Supplementary

# Adsorptive-Oxidative Removal of Sulfides from Water by $MnO_2$-Loaded Carboxylic Cation Exchangers

**Łukasz J. Wilk *, Agnieszka Ciechanowska and Elżbieta Kociołek-Balawejder**

Department of Industrial Chemistry, Wroclaw University of Economics and Business, 118/120 Komandorska St., 53-345 Wroclaw, agnieszka.ciechanowska@ue.wroc.pl (A.C.); elzbieta.kociolek-balawejder@ue.wroc.pl (E.K.-B.)

* Correspondence: lukasz.wilk@ue.wroc.pl

Received: 15 October 2020; Accepted: 11 November 2020; Published: date

## 1. Equipment and Methods Used for Characterization of Obtained Sorbents

Microscopic examinations were performed using a Hitachi S-3400N scanning electron microscope (SEM) equipped with an energy-dispersive spectrometry (EDS) microanalyzer (4 nm, with backscattered electron-BSE detector).

The infrared spectra were recorded on a Fourier transform using a Bruker Vertex 70 V vacuum spectrometer equipped with an air-cooled deuterated-triglycine sulfate (DTGS) detector (Billerica, MA, USA). For all Fourier transform infrared (FTIR) spectra, the diamond attenuated total reflection (ATR) accessory was used. The spectral data were recorded at the resolution of 4 $cm^{-1}$ with collection of 64 scans and further processed using Bruker OPUS software (Billerica, MA, USA).

X-ray powder diffraction (XRD) patterns were collected by an Ultima IV/Rigaku/2008 instrument operated at 40 kV and 40 mA with Cu K$\alpha$ radiation at a wavelength of 0.15406 nm (Tokyo, Japan).

The porous characteristics of the materials were determined from the adsorption isotherms for liquid nitrogen at 77 K using Accelerated Surface Area and Porosimetry Analyzer ASAP 2020, 2007, Micromeritics, and based on the intrusion of mercury into their porous structure acquired using an Autopore IV 9500, Micromeritics (Norcross, GA, USA). A nitrogen adsorption technique was applied to determine pore texture in the range of 0.7–50 nm. Mercury porosimetry was used for examination of partially complementary and wider pore structure, specifically in the range of dimensions from 3 to 600 nm.

The total concentration of Mn in the structure of obtained hybrid materials was determined by inductively coupled plasma atomic emission spectroscopy, ICP-AES iCAP 7400, Thermo Scientific (Waltham, MA, USA).

## 2. Equipment, Methods and Procedures Used during Preparation of Sulfide Solutions and Adsorption Experiments

Adsorption experiments were performed using a batch regime, in a nitrogen atmosphere, by mixing the sorbent in the air-dry condition with 100 $cm^3$ of $Na_2S{\cdot}9H_2O$ (Sigma-Aldrich) in deoxygenated distilled water with concentrations of 100, 200 and 500 mg $S^{2-}/dm^3$. For preliminary tests, 0.50 g of the sorbent was used, and the kinetic chemisorption studies were performed using 0.30 g of the sorbent. The concentration of dissolved oxygen was measured using a WTW Multi 3410 multimeter equipped with an FDO 925 optical probe (WTW, Weilheim, Germany). Nitrogen (6.0, Messer) was inserted into glass stoppered conical flasks with hybrid material and sulfide solution at the initial stage and after collection of each sample.

To quantify possible loss of sulfides in the form of $H_2S$ caused by insertion of nitrogen and sampling, their concentration was also measured in constantly sealed control samples with and without the sorbent.

The products of the sulfide removal process were also identified and quantified in the constantly sealed control samples with examined sorbent. All experiments were carried out at least in two repetitions.

**3. Equipment and Methods Used for Analysis of Spent Sorbent Beads and the Aqueous Phase of the Examined Systems**

The concentration of sulfides was measured using a UV-Vis Specord 210 PC spectrophotometer (Analytik Jena, Jena, Germany) with *N*,*N*-dimethyl-*p*-phenylene sulfate (Sigma-Aldrich). Absorbance measurements were taken at 660 nm.

Concentrations of $SO_3^{2-}$, $SO_4^{2-}$ and $S_2O_3^{2-}$ were determined using a Merck-Hitachi high-performance liquid chromatograph (HPLC, Merck-Darmstadt, Germany, Hitachi-Tokyo, Japan) equipped with a conductometric detector (without suppression), Knauer Smartline 1000 gradient pump (Berlin, Germany) and Shodex IC-I-524A column (Showa Denko, Tokyo, Japan). The analyses were performed at 50 °C using 0.25 mM *p*-hydroxybenzoic acid (Sigma-Aldrich) and 1.2 mM *N*,*N*-diethylethanolamine (Sigma-Aldrich) aqueous solution with 10% v/v methanol (Sigma-Aldrich, Chromasolv for HPLC) as the mobile phase. The flow rate of the eluent was 1.5 $cm^3$/min. The mobile phase was prepared with deionized water (18.3 MΩ/cm, Barnstead, EasyPure RF).

Total sulfur content adsorbed in the HIX structure was determined by the Eschka mixture (Fluka) method according to the ISO 334:1992 (E) standard.

The potential manganese content in the solution—(1) as the overall quantity released from the sorbent beads, (2) Mn bound in sediment left in the aqueous phase (3) and as soluble manganese compounds—was examined using atomic absorption spectrometry with a graphite tube (AAS Avanta). The overall quantity of Mn was analyzed after adding 6 M $HNO_3$ (Stanlab, Poland) to the samples and as a result dissolution of the sediment (if there was any). The soluble manganese compounds were analyzed after separation of the sediment by filtration (HPLC nylon syringe filter, 0.2 μm, La-Pha-Pack) and adding 6 M $HNO_3$. The difference between the overall and soluble manganese quantity was manganese bound in the sediment (or particles greater than 0.2 μm).

**All the chemical reagents used in the described study were of analytical grade.**
